# Trojan pH-Sensitive Polymer Particles Produced in a Continuous-Flow Capillary Microfluidic Device Using Water-in-Oil-in-Water Double-Emulsion Droplets

**DOI:** 10.3390/mi13060878

**Published:** 2022-05-31

**Authors:** Ane Larrea, Manuel Arruebo, Christophe A. Serra, Victor Sebastián

**Affiliations:** 1 Instituto de Nanociencia y Materiales de Aragón (INMA), CSIC-Universidad de Zaragoza, 50009 Zaragoza, Spain; larrea@unizar.es (A.L.); arruebom@unizar.es (M.A.); 2 Department of Chemical Engineering, Campus Río Ebro-Edificio I+D, University of Zaragoza, C/Poeta Mariano Esquillor S/N, 50018 Zaragoza, Spain; 3 Networking Research Center on Bioengineering, Biomaterials and Nanomedicine, CIBER-BBN, 28029 Madrid, Spain; 4 Université de Strasbourg, CNRS, ICS UPR 22, F-67000 Strasbourg, France; 5 Laboratorio de Microscopías Avanzadas, Universidad de Zaragoza, 50018 Zaragoza, Spain

**Keywords:** microfluidics, PLGA, oral administration, Trojan particles, emulsion-solvent evaporation, enteric coatings

## Abstract

A facile and robust microfluidic method to produce nanoparticle-in-microparticle systems (Trojan systems) is reported as a delivery vector for the oral administration of active pharmaceutical ingredients. The microfluidic system is based on two coaxial capillaries that produce monodisperse water-in-oil-in-water (W/O/W) double emulsions in a highly controlled fashion with precise control over the resulting particle structure, including the core and shell dimensions. The influence of the three phase flow rates, pH and drying process on the formation and overall size is evaluated. These droplets are then used as templates for the production of pH-sensitive Trojan microparticles after solvent evaporation. The shell of Trojan microparticles is made of Eudragit^®^, a methacrylic acid-ethyl acrylate copolymer that would enable the Trojan microparticle payload to first pass through the stomach without being degraded and then dissolve in the intestinal fluid, releasing the inner payload. The synthesis of the pH-sensitive Trojan microparticles was also compared with a conventional batch production method. The payloads considered in this work were different in nature: (1) fluorescein, to validate the feasibility of the polymeric shell to protect the payload under gastric pH; (2) poly(D,L-lactic acid/glycolic acid)-PLGA nanoparticles loaded with the antibiotic rifampicin. These PLGA nanoparticles were produced also using a microfluidic continuous process and (3) PLGA nanoparticles loaded with Au nanoparticles to trace the PLGA formulation under different environments (gastric and intestinal), and to assess whether active pharmaceutical ingredient (API) encapsulation in PLGA is due efficiently. We further showed that Trojan microparticles released the embedded PLGA nanoparticles in contact with suitable media, as confirmed by electron microscopy. Finally, the results show the possibility of developing Trojan microparticles in a continuous manner with the ability to deliver therapeutic nanoparticles in the gastrointestinal tract.

## 1. Introduction

Oral administration of active pharmaceutical ingredients (APIs) is the preferable administration route compared to some other routes, such as using parenteral forms, due to different aspects of convenience including: high patient compliance, cost-effectiveness, no requirement of specific sterile conditions and ease of large-scale manufacturing of many oral dosage forms. It is estimated that 84% of the best-selling pharmaceutical products are designed for oral administration, being valued at USD 35 billion with a remarkable annual growth rate of 10% [1,2]. However, APIs administered orally can suffer from low bioavailability if the vector is not well suited to the acidic and biological environments (enzymatic digestion) in the gastrointestinal tract and during the presystemic metabolism before reaching the targeted sites [3]. The most popular dosage forms for oral administration include tablets, capsules, granulates and syrups. However, some of these formulations are not able to circumvent the plethora of harsh conditions and barriers required to reach the target. The development of polymer chemistry has resulted in the design of a wide variety of new polymers with specific functions to address previous shortcomings. For instance, many of the APIs sensitive to acidic pH conditions are formulated with an entering coating. This coating consists of a gastro-resistant polymeric layer, mostly derived from cationic polymers which are stable at acidic pH values to preserve the APIs from degrading in the stomach. The coating is gradually hydrolyzed upon reaching the small intestine, where the pH level is more alkaline than in the stomach. Other polymers have mucoadhesive and mucus-penetrating properties to achieve site-specific internalization or are just designed to be metabolized selectively by the colonic microbiota, meanwhile being resistant to enzymatic digestion in the small intestine [4].

Microparticles and nanoparticles are considered the most promising carriers in drug delivery, being also incorporated downstream into different pharmaceutical dosage forms used in oral administration. Both entities have the potential to improve the stability and solubility of encapsulated cargoes. Differently from nanoparticles, microparticles act only locally since they do not traverse into biological barriers (low intracellular uptake), but enable the loading of large doses of drug cargoes [5] and even nutraceuticals (i.e., probiotics) [6]. Micro/nanomization of APIs enables one to attain a controlled or sustained release pattern to diminish the dosage frequency while the maximum therapeutic effect is achieved. Regarding therapeutic microparticles, a large number of pharmaceutical forms composed of microparticles has been successfully commercialized, mainly using parenteral forms of administration [7] (Trelstar^®^, Sandostatin LAR^®^, Risperdal Consta^®^, Nutropin^®^, Vivitrol^®^, Zilretta^®^, Bydureon^®^, Zmax^®^, Arestin^®^, DepoCyt^®^, etc.). Some FDA-approved drugs are orally administered as microparticles: Cotempla XR ODT^®^, Adzenys XR-ODT^®^, Adzenys ER^®^, Quillivant XR^®^, QuilliChew ER^®^, Aptensio XR^®^. On the other hand, therapeutic nanoparticles are considered when the APIs must be delivered through biological barriers, improving bioavailability, circulation time, safety and absorption. Lipid and polymer-based nanoparticles are the most relevant FDA-approved nanomedicines that are currently used in the clinic for parenteral administration [8]: Doxil^®^, Onivyde^®^, Onpattro^®^, Abraxane^®^, Adynovate^®^, etc.

The most relevant methods developed during the last two decades to produce micro- and nanoparticles with controlled characteristics can be summarized as [2,5,9]: spray-drying, extrusion, coacervation, freeze-drying, emulsification and nanoprecipitation, with all of them using both batch and microfluidic reactors. Combining microparticles and nanoparticles in a single carrier to synergically exploit their potential advantages has resulted in a new concept of delivery systems named nanoparticle-in-microparticle, also known as Trojan systems [10]. Ideally, once orally administered, the microparticle provides protection to the nanoparticles until they deliver their cargo near the targeted site. Once the delivery is activated, the microparticle will dissolve or erode, triggering the release of the inner nanoparticles. On the other hand, nanoparticles will be internalized across different biological barriers, including the intestinal barrier, to release the APIs, minimizing the cargo clearance before reaching the target. Trojan particles are usually produced by the combination of complex multistep procedures [10] including some of the aforementioned for the production of their single counterparts. Nanoparticles are first produced and then microencapsulated without affecting previous specifications, which is sometimes challenging. Generally, spray-drying and gelation techniques are the procedures considered the most, but size control and loading selectivity are not well realized. Each manufacturing approach has pros and cons; the most limiting one is the productivity. In fact, the failure of the majority of formulations to meet FDA Current Good Manufacturing Practices (cGMPs) is related to the complexity of the production process and the lack of reproducibility [11]. As a result, they are plenty of formulations produced at lab scale, but the number is drastically diminished when the production is scaled-up to market. Out of the aforementioned approaches, microfluidics is one of the most apt approaches for producing a plethora of formulations, including nanoparticles [12], microparticles [7], and Trojan particles [13], with an excellent control concerning formulation specifications (size, chemical composition and shape), productivity and reproducibility while maintaining a continuous production.

Micron-sized channels enable one to handle a large number of inlet streams with precision, leading to a highly reproducible mixing of reagents. In addition, microfluidics provides some advantages against classical production systems [14]: portability, low energy consumption, fast screening of synthesis conditions, highly integrated multifunction and easy scalability. It has been accepted that droplet-based microfluidic devices are unique systems for controlling size, shape and loading during the microencapsulation process [15], being also feasible in terms of scaling up the production by a parallelization approach [16]. Previous microfluidic systems based on drop flow have been successfully applied to produce Trojan microparticles. For instance, Liu et al. [17] prepared porous silicon microparticles (c.a. 5 μm) containing two chemotherapeutic drugs. These microparticles were then microencapsulated using a single O/W drop microfluidic system and a pH-responsive polymer (derivative of hypromellose acetate succinate) solubilized in ethyl acetate. The solvent evaporation of ethyl acetate after microdrop formations resulted in polymer precipitation and the production of 129 ± 2.3 μm Trojan particles. Using a similar microfluidic system, Zhang et al. [18] considered a cargo composed of porous silicon nanoparticles (151.1 ± 4.0 nm) functionalized with poly(methyl vinyl ether-co-maleic acid) to endow the cargo with mucoadhesive properties. Then, the cargo was microencapsulated in pH-responsive hydroxypropyl-methylcellulose acetate succinate, yielding homogenous Trojan microparticles with a narrow size distribution (ca. 30 μm). Ideally, nanoparticles should also be produced in continuous flow to produce downstream the Trojan particles, but this approach is challenging and it is highly dependent on nanoparticle purification, concentration, and fluid dynamics. For instance, viscosity, flow rates and surfactant concentration are some key variables that should be carefully studied to achieve a stable drop flow regime. The values of these variables are difficult to match when two different microfluidic processes are coupled, hindering the continuous production. Semi-continuous approaches have been considered in order to overcome previous shortcomings and ease the continuous production of Trojan particles. Our research teams have much experience in the production of nanoparticles, microparticles and Trojan particles using microfluidics. Trojan particles loaded with ketoprofen nanoparticles were successfully produced using UV-induced free radical polymerization [13] in a two-step semi-continuous process. First, a single nanoemulsion composed of ketoprofen, monomers (acrylate and acrylamide) and a photoinitiator was produced with an elongational-flow micromixer. Afterwards, the nanoemulsion was injected into a coaxial capillary-based microfluidic device to generate O/W microdroplets. The microdroplets and their cargo (i.e., nanoemulsion) were exposed to UV irradiation in a continuous flow to activate a dual free radical polymerization to solidify the droplets into Trojan particles with tuneable sizes (200–388 mm) [13,19]. On the other hand, a two-step semi-continuous process was considered to produce thermoresponsive Trojan microparticles composed of poly(N-isopropylacrylamide)-based microparticles loaded with plasmonic hollow gold nanoparticles and bupivacaine [7]. Plasmonic Au-hollow nanoparticles (40 ± 3.2 nm) with NIR absorption were successfully produced under continuous flow using a galvanic replacement reaction and an oxygen segmented flow [20]. The resulting plasmonic nanoparticles were then injected into a coaxial capillary microfluidic device, together with the monomer (*N*-isopropylacrylamide) and the crosslinker (*N*,*N*-methylenebisacrylamide), constituting the aqueous dispersed phase. The continuous phase was composed of hexane, a surfactant (Span^®^ 80) and the photoinitiator (2-diethoxyacetophenone). The flow focus of both phases yielded drops with tunable sizes and, downstream, the reactor was linked to a capillary exposed to UV-LED to activate the radical polymerization and the formation of Trojan microparticles with NIR-light response [7].

This work makes the first attempt to produce pH-sensitive Trojan microparticles with a shell made of Eudragit^®^, a methacrylic acid-ethyl acrylate copolymer that would enable the Trojan microparticle payload to first pass through the stomach without being dissolved and then dissolve in the intestinal fluid, releasing the inner payload. A two-step semi-continuous process based on two coaxial capillaries was designed to produce monodisperse water-in-oil-in-water (W/O/W) double emulsions in a highly controlled fashion in dripping mode, with a precise control over the structure, such as the core and shell dimensions. In order to study the optimal synthesis conditions and achieve the maximum encapsulation efficiency, the influence of the following variables on the generation of the Trojan microparticles was studied: (1) continuous phase and surfactant viscosity; (2) capillaries’ alignment and their relative position; (3) system pH; (4) washing, drying and storage of the microparticles; (5) Eudragit^®^ polymer concentration; and (6) continuous and internal phase flow rates. The synthesis of the pH-sensitive Trojan microparticles was also compared with a conventional batch production method. Finally, the gastro-resistant behaviour of the produced Trojan MPs was evaluated using a novel procedure based on the use of PLGA nanoparticles loaded with Au nanoparticles which have the role of being tracers and make their identification by electron microscopy analysis easier.

## 2. Experimental Section

### 2.1. Materials

Both polymers—Eudragit^®^ L100-55, an enteric polymer based on an anionic methacrylic acid-ethyl acrylate copolymer (1:1), and Resomer^®^ RG 504, an ester terminated poly(D,L-lactic acid/glycolic acid) 50:50 (PLGA; MW 38–54 KDa)—were supplied by Evonik Industries (Evonik Röhm GmbH, Germany). The surfactants sodium cholate hydrate (<99%), Pluronic F68, Span^®^ 80 and PVA (Mowiol^®^ 18–88, P.M~130,000); the encapsulated drug rifampicin, fluorescein 5-isothiocyanate (FITC); and methylcellulose and solvents including ethyl acetate, ethanol, acetonitrile were purchased from Sigma Aldrich (St. Louis, MO, USA) and used as received.

### 2.2. Nano- and Microparticle Synthesis

#### 2.2.1. PLGA-Based NPs Produced in Continuous Flow

PLGA-based nanoparticles were prepared by two different procedures published by our group elsewhere [21,22,23] and here briefly summarized. Rifampicin-loaded PLGA nanoparticles were prepared in a continuous PEEK-made interdigital micromixer (SIMM-V2, Slit Interdigital Micro Mixer, IMM, Mainz, Germany) using an oil-in-water (O/W) emulsification process followed by a solvent evaporation procedure [21,22]. The organic phase was composed by 1% (*w*/*v*) PLGA (50:50) polymer, 0.1% (*w*/*v*) rifampicin, 2% (*w*/*v*) surfactant (Pluronic F68) and ethyl acetate (used as organic solvent). Aqueous and organic phases were injected by syringe pumps (Harvard Apparatus) at the proper flow rate to achieve a residence time of 10 ms. Ethyl acetate was evaporated after the emulsification process to yield rifampicin-PLGA (PLGA-RIF) nanoparticles. On the other hand, Au-PLGA nanoparticles were produced in two consecutive PEEK-made interdigital micromixers (SIMM-V2, Slit Interdigital Micro Mixer, IMM, Mainz, Germany) using a double emulsion water-in-oil-in-water (W/O/W) emulsification process followed by a thermal treatment to activate the Au precursor reduction and the solvent evaporation [13,23]. A primary W/O emulsion was produced after mixing in continuous flow an aqueous stream consisting of chloroauric acid and sodium citrate, with an organic stream of ethyl acetate and PLGA. The resulting emulsion was injected under continuous flow in a second micromixer, together with an aqueous stream consisting of sodium cholate to form a double W/O/W emulsion. Afterwards, the Au precursor loaded in the internal phase was heated at 45 °C in a continuous flow with a residence time of 10 min to promote electron supply from sodium citrate (redox reaction) forming Au nanoparticles. Finally, the organic solvent (ethyl acetate) was evaporated under continuous stirring in an open flask to yield Au-PLGA NPs. The synthesis of these nanomaterials was carried out with the Synthesis of Nanoparticles Unit of the ICTS “NANBIOSIS” at the Institute of Nanoscience and Materials of Aragon (INMA) Universidad de Zaragoza.

#### 2.2.2. Trojan Eudragit^®^ Microparticles Produced in a Batch Type Reactor

Trojan Eudragit^®^ microparticles were prepared in a batch type reactor using a W/O/W double emulsification process with solvent evaporation. This procedure was published elsewhere by our group [3] and is here briefly described. The method used rifampicin-loaded PLGA–NPs in the inner water phase. In a mixture of ethanol:ethyl acetate (1:4; organic phase), 2% (*w*/*v*) Eudragit^®^ L100-55 was dissolved and emulsified with a PLGA-RIF suspension by ultrasonication (Digital Sonifier 450) at a 40% amplitude for 30 s. The formed W/O emulsion was then emulsified with an aqueous solution of sodium cholate at 40% amplitude for 35 s to obtain the W/O/W emulsion. To promote the stability of the final emulsion, 0.3% (*w*/*v*) sodium cholate solution was also added before the solvent evaporation step. The organic solvent was evaporated under continuous stirring to obtain Trojan Eudragit^®^ microparticles loaded with PLGA-RIF nanoparticles.

#### 2.2.3. Trojan Eudragit^®^ Microparticles Produced in Continuous Flow

**Figure 1:**
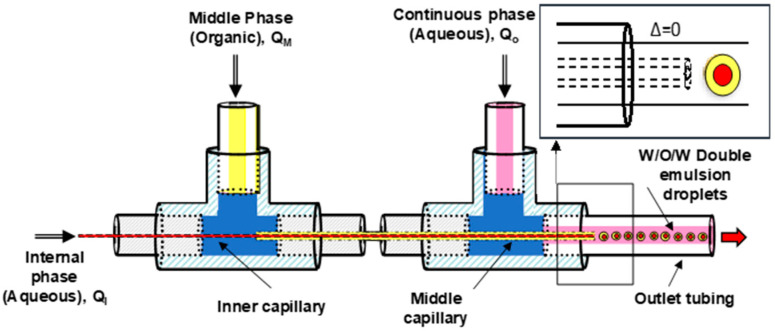
Scheme of the microfluidic system formed by two coaxial capillaries used for the production of Trojan MPs by double emulsion.

The microfluidic system used for the continuous synthesis of pH-sensitive Trojan microparticles (MPs) is based on the microfluidic droplet device developed by Khan et al. for the synthesis of core-shell microparticles sensitive to pH [13], with certain modifications. The microfluidic system consisted of three syringe pumps (Harvard Apparatus), two T-joints (1/16”, Upchurch Scientific), a hydrophilic internal capillary (silica tube with an internal diameter of 100 μm, Polymicro Technologies) that was used to inject the internal aqueous phase and a hydrophobic middle capillary (PEEK tube with 255 μm internal diameter, Upchurch Scientific) that allowed one to administer the middle organic phase. Both capillaries were arranged coaxially and placed in the center of a PTFE outlet tubing (1 mm internal diameter). The two dispersed phases (internal and middle phase) were injected in the continuous phase, forming a double drop at the tip of the coaxial capillaries, as shown in Figure 1. Finally, the organic solvent (ethanol/ethyl acetate) was evaporated from the double emulsion droplets generated in the coaxial system to favor the precipitation of the polymer and the formation of the Trojan MPs.

Due to the high sensitivity of the droplet microfluidic system, the composition of each phase of the emulsion must be carefully selected, as well as the operating conditions used for the formation of the pH-sensitive Trojan MPs. The droplet internal aqueous phase was varied depending on the formulation aim using: fluorescein 5-isothiocyanate as a model to optically analyze the microparticle formation, Au-PLGA NPs to assemble Trojan MPs with a potential application in theragnosis [24] and PLGA-RIF NPs to produce a therapeutic formulation of Trojan particles with a potential use in the oral treatment of mycobacterium tuberculosis [3]. The droplet middle organic phase is composed of an established concentration of enteric polymer (Eudragit^®^ L100-55) previously dissolved in ethanol and 1% (*w*/*v*) Span^®^ 80 dispersed in ethyl acetate, which acts as surfactant. Eudragit^®^ concentration was varied from 2 to 8% (*w*/*v*), maintaining the EtOH:EA ratio of 1:4. Lastly, the continuous phase was constituted by an aqueous solution of methylcellulose with 1% (*w*/*v*) PVA (Mowiol^®^ 18–88, P.M. ~130,000) as surfactant. The concentration of methylcellulose was varied (2, 1, 0.5 and 0.25% *w*/*v*) to tune the viscosity of the continuous phase and to control the dripping mode that modulates drop formation and size.

The three aforementioned phases were injected in the capillary coaxial system described using three syringe pumps with the following flow rates: Q_i_ = 2–5 μL/min, Q_m_ = 10 μL/min and Q_o_ = 200–800 μL/min. After contact at the tips of the two capillaries, the three phases generate the double phase microdroplets, with an aqueous core and a polymeric shell, Figure 1.

Next, the double emulsion drops were collected in a glass flask preloaded with an aqueous solution of PVA (Mowiol^®^ 18–88, MW ~130,000) at 0.3% (*w*/*v*), where they precipitated in the form of polymeric microcapsules after evaporation of the organic solvent by magnetic stirring. Finally, the MPs were washed three times with distilled water to remove the excess of methylcellulose and then dried and stored for further use.

## 3. Characterization

### Droplet and Particle Size Analysis

Droplet formation was monitored by coupling a CCD camera (Pike F-032B, Allied Technology) with a microscope (Eclipse 80i, Nikon). The camera captures up to 200 fps at a full resolution of 648 × 488 pixels. Core diameter and shell thickness of the particles (*N* > 60) and droplets (*N* > 60) were measured using the image analysis module of the software controlling the CDD camera (Hiris, R&D vision, France).

Viscosity of the different phases was measured using an Ubbelohde viscometer at 25 °C.

The morphology and size distribution of the final Trojan microparticles were determined at the Advanced Microscopy Laboratory (LMA-UNIZAR) with an environmental scanning electron microscopy SEM-Quanta FEG-250, without any pre-treatment on the samples. Each experiment was performed at least three times and to obtain the particle size statistics at least *N* > 60 microparticles were considered. PLGA-based nanoparticles were characterized using scanning electron microscopy (SEM, Inspect F50, FEI, Eindhoven, The Netherlands) at an accelerating voltage of 10–15 kV. PLGA-based nanoparticles were immobilized on a silicon chip, stained with phosphotungstic acid hydrate, dried and coated with a platinum layer. At least 150 particles were measured to evaluate the mean diameter and distribution of the particles. On the other hand, PLGA-based nanoparticles were also characterized by transmission electron microscopy in order to increase the resolution on nanoparticle features. The electron microscopy observations were carried out using a T20-FEI microscope with a LaB6 electron source operated at 200 kV. PLGA NPs were also stained with phosphotungstic acid hydrate, pipetted onto a TEM copper grid with a Formvar continuous carbon film. The size of hydrated PLGA-based NPs was also determined with Dynamic Light Scattering (Zeta Plus, Brookhaven Instruments Corporation, Holtsville, NY, USA) after appropriate dilution with Milli-Q water (at least five replicated measurements were performed).

To test the pH-responsive behavior of Trojan MPs, samples were subjected to simulated gastric and intestinal fluids. The dissolution tests were carried out applying the protocols described by Patel et al. (2012) [25] and Sun et al. (2015) [26], with buffer solutions prepared according to the fundamentals provided by the American Pharmacopoeia USP26-NF [27]. Thus, first, the microparticles were exposed to a fluid solution simulating gastric conditions (0.1 N HCl, pH = 1.2) for 2 h at a temperature of 37 °C and then the same microparticles were separated by filtration and transferred to a simulated intestinal fluid solution (0.05 M KH_2_PO_4_, pH adjusted to 6.8 ± 0.1 with 0.2 N NaOH) for 6 hours, keeping the temperature constant at 37 °C.

The determination of the encapsulated NPs in the core of the Eudragit^®^ Trojan MPs was performed as follows: (1) The MPs were dissolved in PBS at pH = 7.4 (over the degradation pH of Eudragit^®^ L100 = 5.5) to release the PLGA-rifampicin NPs; (2) the NPs released were washed by centrifugation to eliminate the traces of enteric polymer and the PBS salts that were added; (3) The washed NPs were analyzed with UV-Vis (Jasco V-670 spectrophotometer). The encapsulation efficiency of PLGA-rifampicin NPs was determined with UV-Vis directly (NPs liberated from Trojan MPs at pH > 5.5), considering the absorbance of the nanoparticles at 477 nm, corresponding to one of the three absorbance peaks characteristic of rifampicin [28]. Once the absorbance was determined from the spectrum of each sample, its encapsulation efficiency was calculated using the linear adjustment obtained from the calibration curve of the starting *NPs* (*PLGA-RIF NPs* used for the encapsulation in the enteric polymer, Figures S1 and S2). The results are expressed in both encapsulation efficiency (% *EE*) and drug loading (% *DL*), using Equations (1) and (2), respectively.

(1) $$EE\;\left(\%\right)=\frac{Amount\;of\;PLGA-RIF\;NPs\;loaded}{Total\;amount\;of\;NPs\;used}\times 100$$

(2) $$DL\;\left(\%\right)=\frac{Amount\;of\;PLGA-RIF\;NPs\;loaded}{Total\;amount\;of\;Microparticles}\times 100$$

## 4. Results and Discussions

The variables that influence the formation of multiple-core drops (W/O/W, O/W/O) in a coaxial microfluidic system are numerous [19,29], but out of them, it can be highlighted that the most relevant are: (1) hydrodynamic conditions, (2) the alignment of capillary tips and their relative position, (3) pH of the external phase, (4) drying process, (5) polymer concentration in the middle phase stream, and (6) internal and continuous phase flow rates.

### 4.1. Hydrodynamic Conditions

**Figure 2:**
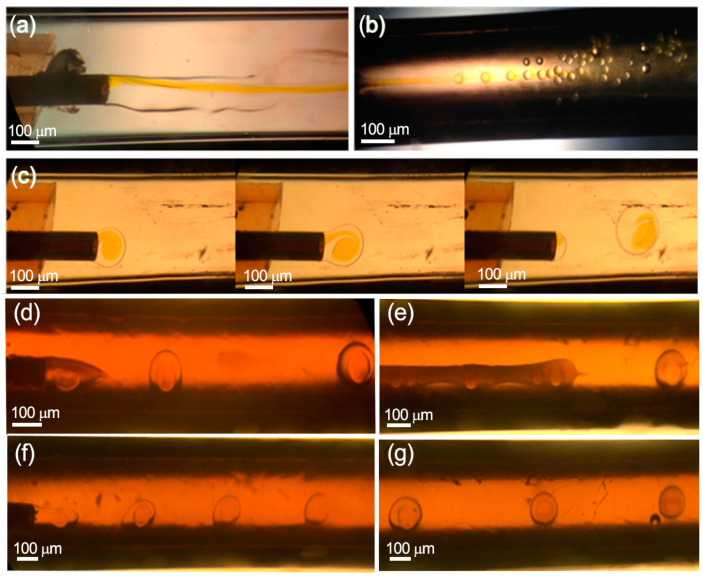
Optical microscopy images of the double drop of emulsion formed at the end of the coaxial capillaries according to the composition of the phases. Without surfactants in the continuous and dispersed phases, 2% Eudragit^®^: (**a**) viscosity of the continuous phase < 500 cSt; (**b**) viscosity of the continuous phase = 500 cSt. Addition of surfactants in the continuous and dispersed phases: (**c**) viscosity 500 cSt, 2% Eudragit^®^; (**d**) viscosity 1000 cSt, 2% Eudragit^®^; (**e**) viscosity 1000 cSt, 4% Eudragit^®^; (**f**) viscosity 2000 cSt, 2% Eudragit^®^; (**g**) viscosity 2000 cSt, 4% Eudragit^®^.

According to the hydrodynamics for multiple-core drop formation [30], the inner and middle injected solutions must be immiscible fluids as well as the middle and outer phases, or at least the different viscosity between both phases must be high enough to reduce molecular diffusion and apparent miscibility between them. On the other hand, the surface energy of the capillaries should be adapted to the fluid wettability. In this case, the aqueous inert fluid was injected through a capillary with a hydrophilic inner surface, whereas the middle capillary surface was hydrophobic. On the other hand, it is necessary to add surfactants into the middle and outer phases to control the interfacial tension forces and to promote a stable and homogenous double drop formation (Figure 2).

Figure 2a shows that a co-flow regime is formed when the continuous phase is injected without surfactants due to the small viscous forces and the high surface tension in the middle interface. In contrast, the increase in viscosity of the continuous phase by adding methylcellulose and the reduction of the interphase surface tension by the addition of PVA as surfactant increased the shear forces exerted on the middle phase and favored the breakup of the middle phase into droplets. A short break time induced by large shear forces can decrease the droplet diameters due to the higher shear stress applied to the dispersed phase [31]. The relation between viscosity and drop rupture is depicted in Figure 2c,d,f, where it is observed that the droplet size is reduced by increasing the viscosity of the continuous phase. As a result, the diameters of the drops were 859.2 ± 28.5 μm, 624.0 ± 31.6 μm and 506 ± 24.9 μm when the viscosity of the continuous phase was varied from 500 to 1000 and 2000 cSt, respectively. However, by increasing the viscosity at 3000 cSt, the diameter of the microparticles did not decrease; the viscosity was so high that the double drops could not be formed. Therefore, there is a maximum value of viscosity (2000 cSt), from which the minimum diameter that can be reached is limited by the internal diameter of the capillary [31] (510 μm). Finally, it can be inferred from Figure 2e,g that if the viscosity of the middle phase was doubled, by increasing the concentration of the polymer, the viscosity of the continuous phase should be proportionally increased to preserve a steady dripping mode.

### 4.2. Capillaries’ Alignment and Their Relative Position

There are different capillary arrangements [29], where the capillaries’ tips can be assembled with similar, positive and negative relative positions (Δ). Single and multiple cores can be engineered depending on the relative position of the inner and middle capillaries’ tips. Taking into account previous results [29], the microfluidic device considered in this work was designed with similar relative position (Δ = 0), where droplet formation was achieved in dripping mode to ensure the formation of monodisperse drops. The inner capillary tip, where the core droplet is generated, was assembled at the same position as the middle capillary tip, where the shell layer is formed, Figure 1.

### 4.3. pH at the External Phase

**Figure 3:**
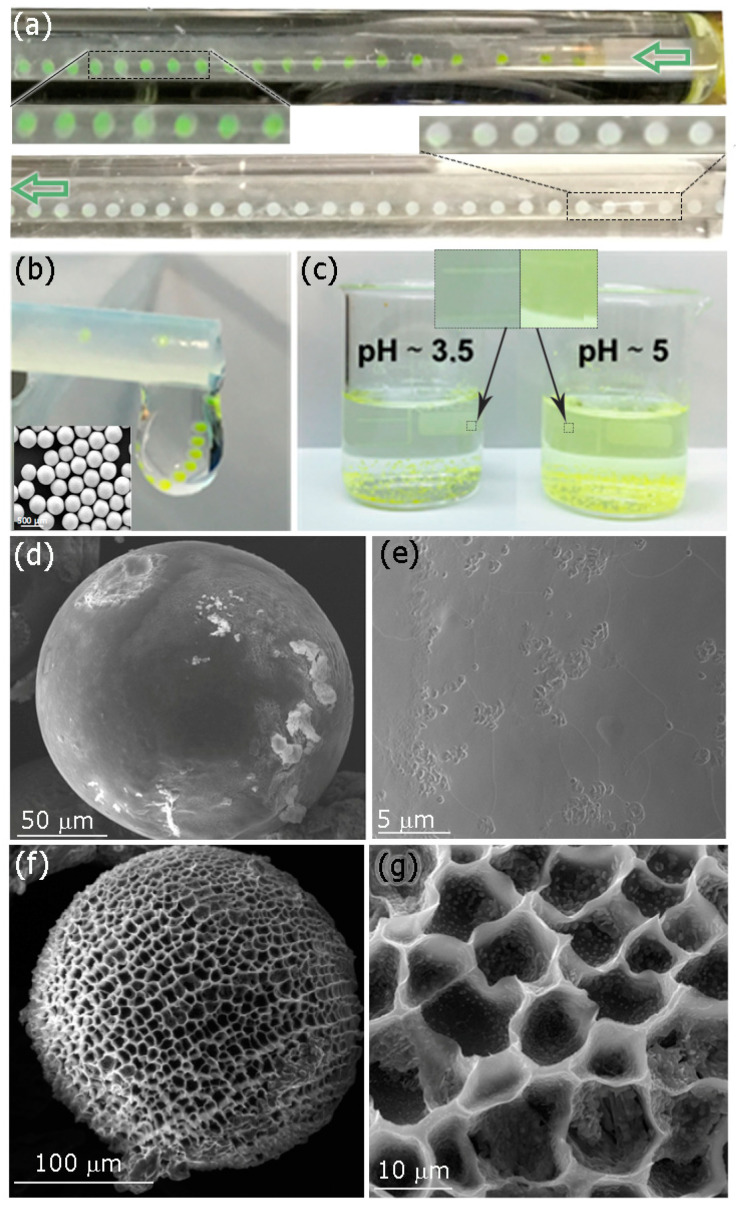
(**a**) Droplets of double emulsion formed at the tip of the coaxial capillaries when the pH of the continuous phase was 6.5 (yellowish) and at the outlet (white); insets depict the drops’ hue at the tip of the capillaries and at the outlet. (**b**) Double emulsion drops stabilized at the outlet of the microfluidic system when the pH of the continuous phase was 3.5. Inset represents a SEM image of produced MPs. Scale bar is 500 μm. (**c**) Comparison of the enteric microparticles formed after the evaporation of the solvent at different pHs (3.5 and 5). Q_i_/Q_m_/Q_o_ = 2/10/200 μL/min at room temperature. The insets show the solution hue. Cryo-SEM images of MPs at different pHs after 24 h incubation: (**d**,**e**) pH = 3.5 and (**f**,**g**) pH = 5.

Due to the enteric nature of the polymer used in this work, with a shell having a specific dissolution pH above 5.5, and considering that APIs are located in the aqueous core, it was important to preserve the double drop structure (W/O/W) from the tapered tip of the coaxial capillaries all the way to the outlet of the microfluidic system and during the solvent evaporation process. Ethyl acetate, the solvent used in the middle phase, is partially soluble in water [12] and this solubility is enhanced by the highest pressure vapor of ethyl acetate at 25 °C (partial pressure driving force of ethyl acetate is 13 kPa at 25 °C). The diffusion of ethyl acetate to the continuous phase promotes the polymer shell crystallization because Eudragit^®^ is not soluble in water and the APIs were isolated from the external environment. Figure 3a shows that the fluorescein loaded in the internal phase, used as a tracer, diffuses easily in the continuous phase once the drop is formed because the pH of the continuous phase is larger than 5.5 (pH = 6.5) and the crystallized polymer degrades quickly before reaching the outlet.

To face this shortcoming, the pH of the continuous phase was decreased to 3.5. Figure 3b depicts the MPs produced under pH = 3.5, where the fluorescein hue is maintained. Consequentially, to preserve the APIs inside the Eudragit^®^ MPs, it is necessary to work at controlled pH conditions, always lower than the dissolution pH of Eudragit^®^ L100-55 (pH < 5.5). Finally, collected MPs loaded with fluorescein were incubated at pH = 3.5 for 24 hours and no fluorescein was released (Figure 3c). SEM images showed a smooth surface with no pores; just a few dimples are formed (Figure 3d,e). On the other hand, fluorescein release was observed when MPs were exposed to a pH solution of 5 for 24 h (Figure 3c). The electron microscopy analysis of MP surface evidenced the presence of pores of approximately 10 μm in diameter that promoted dye diffusion upon deprotonation of their carboxylic acid groups (Figure 3f,g).

### 4.4. Polymer Concentration in the Middle Phase Stream

**Figure 4:**
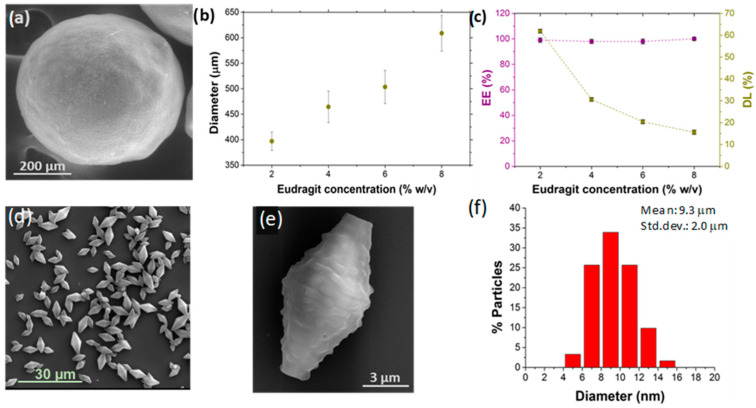
(**a**) SEM image of a Trojan microparticle synthesize with 2% (*w*/*v*) Eudragit^®^ using the coaxial microfluidic system. Study of the influence of Eudragit^®^ concentration on: (**b**) the diameter, (**c**) the encapsulation efficiency (EE) and drug loading (DL) of the enteric microparticles synthesized in the microfluidic system. (**d**,**e**) SEM images and (**f**) size distribution of the Trojan microparticles obtained in batch conditions with 2% (*w*/*v*) Eudragit^®^.

As mentioned, viscosity, viscoelasticity and interfacial tension are hydrodynamic parameters that govern the uniform formation of droplets by using the dripping mode. Regarding the polymer concentration in the middle phase, despite not preventing the collapse of the enteric MPs during their drying process at room temperature, it exerts a great influence on MPs’ size, as well as on their encapsulation efficiency and loading. The increase of the polymer concentration in the middle phase (2–8%), while keeping constant the other operating and material parameters (pH = 3.5, surfactant concentration, flow rates), leads to a progressive increase in the enteric MPs’ size. The size of MPs varied from 397.1 μm ± 17.8 μm to 605 μm ± 29.2 μm when the Eudragit^®^ concentration was increased from 2% to 8% *w/v*, respectively (Figure 4a,b and Figure S2). This tendency is justified because increasing the concentration of Eudragit^®^ polymer in the middle organic phase increases the viscosity of the dispersed phase, decreasing the viscosity difference and the shear stress between the external and middle phases (Figure 4a,b). As a result, there is a retardation of the speed of breakup and drops are larger in size. The coefficient of variation (CV) of polymer MP sizes remains below 5% *w*/*v* for the low polymer concentrations, but at 8% *w*/*v* when the CV is large, this being an indication of polydispersity (Figure 4a,b). This is consistent with previously reported observations when using the dripping mode [19].

Fluorescein was substituted in the internal phase by PLGA NPs loaded with rifampicin (PLGA-RIF NPs). The overall MP diameter was not influenced by the different APIs dispersed in the internal phase. This is probably caused because the shear stress at the outer/middle interface remains almost invariant to the inner phase [29]. MPs loaded with PLGA-RIF NPs were lyophilized to preserve the APIs during long-term storage. Lyophilized MPs were incubated at pH = 7.4 to dissolve the enteric shell and release the PLGA-RIF NPs. This process eases the determination of encapsulation efficiency and loading by measuring the absorbance of the PLGA-RIF NPs at λ = 477 nm by UV-Vis spectrophotometry As depicted in Figure 4c, the coaxial microfluidic system enabled the achievement of 100% EE, maintaining this percentage at all the polymer concentrations tested. This result was granted by the fast precipitation of the polymeric shell, which is promoted by the acid nature of the external phase (pH = 3.5). Consequently, the internal phase is totally wrapped by the middle phase and no diffusion occurs between the internal and external phases. On the other hand, despite the high EE reached, the percentage of drug loading (DL) decreased gradually while increasing the concentration of Eudragit^®^ in the formulation of MPs (Figure 4c). Since the concentration of the drug added in the internal phase remains constant in all the tests carried out, this result is rationalized by the thicker polymeric shell resulting in a small internal core as the polymer concentration is increased. Therefore, we selected 2% (*w*/*v*) Eudragit^®^ as the optimal polymer concentration in the middle phase to obtain a complete EE (~100%) and the highest DL (62.5%).

When comparing the Trojan MPs obtained using the microfluidic system (Figure 4a) with the ones obtained by the batch type reactor (Figure 4d,e), significant differences could be observed: (1) SEM morphological analysis revealed spherical particles with micrometric sizes (397.1 ± 17.8 μm) in the coaxial system, compared to the bipyramidal structure with smaller sizes (9.3 ± 2.0 μm, CV ~21%) when using batch reactors. (2) The maximum EE obtained by the batch was 48.0 ± 2.7%, corresponding to 9.6 ± 0.5% DL, compared to a 100% EE and a DL of 62.5 ± 2.0% obtained in the microfluidic system, both with 2% *w/v* Eudragit^®^ concentration during the production process. These differences are remarkable and highlight that the coaxial microfluidic system considered in this work is more efficient in terms of loading different APIs than the conventional W/O/W emulsification processes produced in a batch-type reactor. In addition, the microfluidic system can be continuously operated with high reproducibility and saves a costly polymer.

### 4.5. Internal and Continuous Phase Flow

**Figure 5:**
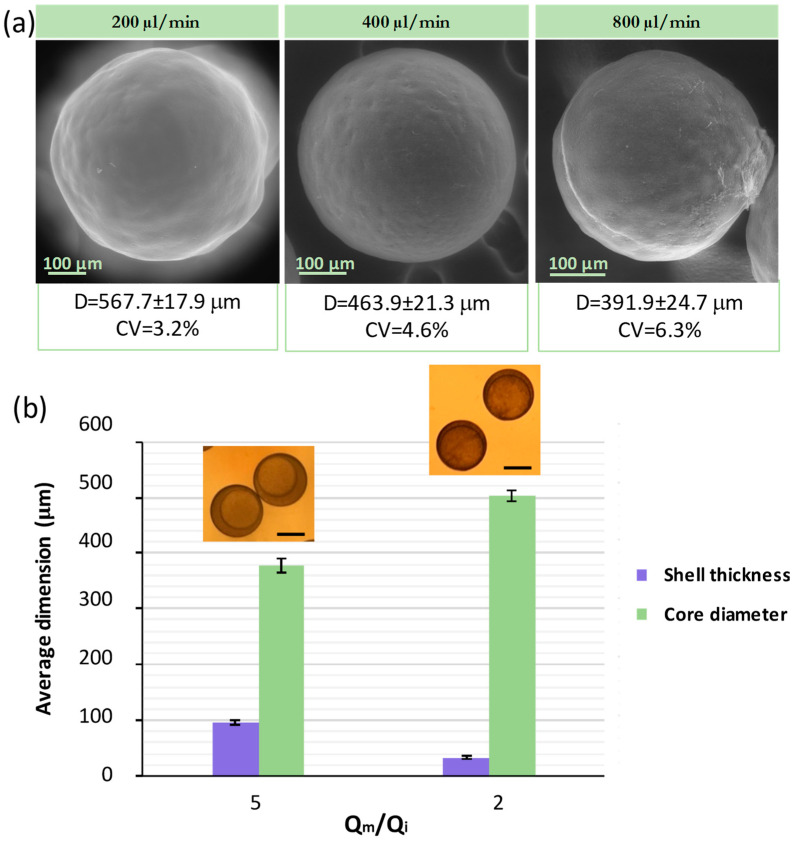
(**a**) SEM image of Trojan microparticles produced by altering the continuous phase flow rate (Qo) from 200 to −800 μL/min and leaving constant the internal (Qi, 5 μL/min) and middle flow rates (Qm, 10 μL/min). Mean size and coefficient of variation (CV). (**b**) Effect of Qm/Qi ratio when Qo (200 μL/min) and middle flow Qm (10 μL/min) were kept constant. Shell and core diameters were measured from optical images such as the ones depicted in the insets.

In the process of generating W/O/W double emulsion droplets by a microfluidic drop generator, the flow ratio of the three-phase liquid has significant influence on the flow pattern and the resulting droplets. In particular, the ratio between the continuous and middle flow Qc/Qm governs the MP size. Generally, the droplet size decreases while increasing the continuous phase fluid rate in dripping mode flow patterns [29]. In this case, the droplet generator was operated at relatively low velocity for all fluids and the W/O/W droplets were formed in the dripping mode, which also ensures good control during drop formation as well as high droplet size monodispersity. To study the influence of the continuous flow on the droplet size, the continuous flow (Qo) was varied from 200 to 400 and to 800 μL/min while the internal (Qi, 5 μL/min) and middle flows (Qm, 10 μL/min) were kept constant, as well as previously studied variables. This implies that the Qo/Qm ratio was varied from 20, 40 to 80, respectively. The MPs produced with Qo/Qm = 20 were the largest, yielding to a mean size of 567.7 μm and a CV of 3.2%. When the Qo/Qm ratio was increased to 40, the MP size was decreased to 463.9 μm with CV = 4.6% (Figure 5a).

Finally, an MP mean size of 391.9 μm and a CV of 6.3% were obtained when the Qc/Qm ratio was increased to 80 (Figure 5a). These results are rationalized to be caused by droplet formation while requiring a balance between interfacial tension and the viscous drag force exerted by the continuous sheath flow. In these flow conditions, the drag force increases as the continuous phase velocity does and the larger shear stress exerted by the continuous phase promotes a fast pinch out of the droplet before it grows [7]. Consequently, the results obtained confirm that the MPs’ size increases as the flow rate of the continuous stream is decreased, while middle and inner flows are kept constant.

The effect of changing the internal aqueous phase was studied while the continuous flow (Qc, 200 μL/min) and middle flow (Qm,10 μL/min) were kept constant. Figure 5b shows that the overall droplet size is similar, while the core droplet diameter increases as the Qm/Qi ratio decreases from 5 to 2, resulting in core droplet sizes of 376.7 μm and 503.6 μm, respectively. In other words, the core size increases and the shell size decreases as the internal flow rate increases. In all systems tested under the same Qc/Qm, it was observed that the core and the overall core-shell drop had the same formation time. Consequently, the overall droplet diameter was not influenced by the inner flow as it remained constant while increasing the inner fluid flow rate. This is in agreement with the literature [29], and it is ascribed to the fact that the shear stress at the outer/middle drop interface remains almost the same when the inner fluid flow rate is varied.

### 4.6. Drying Process

**Figure 6:**
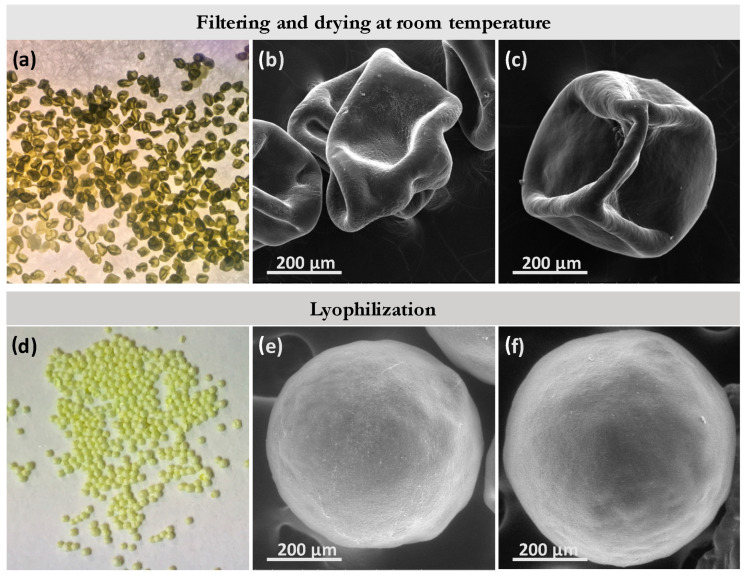
Drying of the enteric microparticles synthesized in the coaxial microfluidic system, Q_i_/Q_m_/Q_o_ = 5/10/200 μL/min. (**a**) Optical microscopy image of the 2% Eudragit^®^ microparticles after filtering and drying at room temperature. SEM images of the microparticles synthesized with (**b**) 2% and (**c**) 4% Eudragit^®^ after filtering and drying at room temperature. (**d**) Optical microscopy image of the 2% Eudragit^®^ microparticles after the lyophilization process. SEM images of the microparticles synthesized with (**e**) 2% and (**f**) 4% Eudragit^®^ after the lyophilization process.

The W/O/W structure of MPs must be designed with an internal core large enough to promote a high API loading. To fulfill this requirement, the polymeric shell should be as thin as possible to promote the APIs’ insulation from the external media and to ensure economic feasibility, since pH-sensitive polymers are costly. However, the shell thickness requirement should also be consistent with the droplet mechanical stability to withstand surface collapse during the drying process. In this case, MPs collapsed after being dried at room temperature, regardless of the polymer concentration (4% *w*/*v*, 6% *w*/*v*, and 8% *w*/*v*) used in the middle organic phase (Figure 6a–c). This phenomenon can be rationalized by the drying rate, where a high drying rate promotes the quick formation of a dry crust [32]. If the vapor pressure is high because of using low boiling point solvents (i.e., ethyl acetate), the crust cannot withstand the internal mechanical forces exerted by the solvent during evaporation and the spherical structure collapses and forms dimples. Consequently, the key point is to control the internal vapor pressure during the drying process. Considering that in this case at room temperature the MP collapse was evident, a low temperature dehydration process was proposed. MPs were frozen in liquid nitrogen and lyophilized to slowly remove the solvent by ice sublimation. Proceeding in this way, the structural stability of the dry particles was successfully achieved, retaining their spherical morphology (Figure 6d–f).

Considering the results obtained in each of the variables tested, the optimal conditions of composition and operation are the following: (1) viscosity of the continuous phase of 2000 cSt, corresponding to a concentration of methylcellulose of 1% (*w*/*v*); (2) surfactant Span^®^ 80 in the middle phase and PVA in the continuous phase, both with a 1% (*w*/*v*) concentration; (3) system pH, continuous phase and evaporation solution at pH = 3.5 ± 0.5; (4) washing, drying and storage of the microparticles. Wash the microparticles with an aqueous solution at pH = 3.5 ± 0.5 and subsequent lyophilization; (5) polymer concentration, 2% (*w*/*v*) since a low polymer concentration carries a higher inner load percentage; (6) Continuous phase flow rate. The best balance between the monodispersity of the particles (CV < 5%) and their size was obtained when using a flow rate of 400 μL/min; (7) internal phase flow rate, 5 μL/min, assessing the fact that the larger the size of the core in the emulsion drop, the greater the volume of the active load encapsulated.

**Figure 7:**
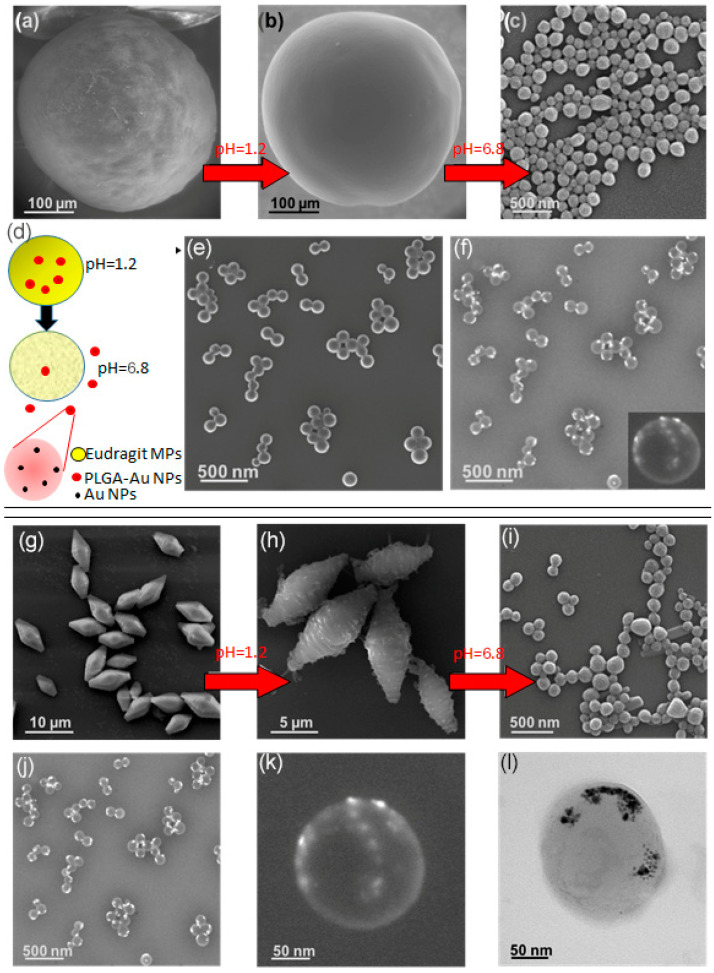
Trojan Eudragit^®^ microparticles synthesized using the coaxial microfluidic system and loaded with PLGA-RIF and PLGA-Au NPs: (**a**) SEM image of the Eudragit^®^ microcapsules loaded with PLGA-RIF NPs; (**b**) SEM image of the microcapsules loaded after 2 h of contact with the simulated gastric fluid, pH = 1.2 and Tª = 37 °C; (**c**) SEM image of PLGA-RIF NPs delivered after dissolution of the Eudragit^®^ microcapsules in the simulated intestinal fluid, pH = 6.8 for 6 h at 37 °C; (**d**) schematic description of the evolution of Trojan microparticles with pH; (**e**) SEM image (secondary electrons) of released PLGA-Au NPs at pH = 6.8; (**f**) SEM image (backscattered electrons) of released PLGA-Au NPs at pH = 6.8. Trojan Eudragit^®^ microparticles synthesized using the batch method: (**g**) SEM image of the Eudragit^®^ microcapsules loaded with PLGA-RIF NPs; (**h**) SEM image of the microcapsules loaded after 2 h of contact with the simulated gastric fluid; (**i**) SEM image of PLGA-RIF NPs delivered after dissolution of the Eudragit^®^ microcapsules in the simulated intestinal fluid; (**j**,**k**) back-scattered electron micrograph to show the location of Au NPs inside the PLGA NP; (**l**) TEM image of a zoomed Au-loaded PLGA NP.

To corroborate the enteric character of the Trojan microparticles synthesized with microfluidic (Figure 7a) and batch methods (Figure 7g), we subjected the resulting particles to a gradual variation of pHs, see the scheme in Figure 7d. Initially, the microparticles were exposed for 2 hours to a solution of simulated gastric fluid (pH = 1.2), acquiring a less rough surface appearance, but keeping its structure mostly unaltered, Figure 7b,h. Next, the same microparticles were transferred to a simulated intestinal fluid solution (pH = 6.8 ± 0.1) where they were maintained for 6 h at 37 °C. Eudragit^®^ capsules dissolved rapidly in contact with the intestinal fluid, boosting the release of the inner encapsulated PLGA-RIF nanoparticles, Figure 7c,i. We assume that the nanoparticles observed by SEM analysis after the simulated intestinal treatment were PLGA-RIF because the particle size (215 ± 65 nm) was similar to the ones loaded during the production of W/O/W MPs (210 ± 63 nm), but it could also happen that the observed nanoparticles were just recrystallized Eudragit^®^ nanoparticles.

To confirm if Eudragit^®^ could recrystallize into nanoparticles, PLGA-RIF NPs were replaced by PLGA-Au NPs in the stream used as the internal aqueous phase during the droplet production process. The interesting fact of using PLGA-Au NPs as a tracer is that the microfluidic approach followed to produce them assures excellent control concerning the Au payload and each PLGA NP contains several Au nanoparticles. Then, the high atomic contrast of Au should be enough to elucidate by back-scattered electron SEM imaging whether the NPs observed after exposing them to the simulated intestinal fluid solution were either the load of Trojan MPs or recrystallized Eudragit^®^. The gastro-resistant behavior of these Trojan MPs, either produced by batch or by continuous approaches, is analogous to that of the Trojan MPs loaded with PLGA-RIFA, maintaining their structure at gastric pH. Similarly to PLGA-RIF, PLGA-Au NPs were released from the MPs produced with both synthetic approaches at intestinal pH, as can be observed in Figure 7e,j. The presence of gold in the matrix of the released PLGA NPs was confirmed by back-scattered electron analysis, where the presence of high contrast due to the location of Au NPs (Figure 7f,j) is observed. For instance, the SEM images depicted in Figure 7e,f are similar, but Figure 7f is a result of elastic collisions of electrons with atoms, where large atoms such as gold induce a stronger electron scattering than carbon atoms from PLGA, providing a conclusive confirmation that Au NPs are loaded.

Figure 7k,l presents TEM images of a representative PLGA-Au NP released from the Trojan MPs, depicting the presence of Au NPs with a size smaller than 15 nm. Consequently, the results presented in Figure 7 strengthen the idea that the enteric Trojan MPs synthesized in this work prevented the degradation at gastric pH of the active compounds encapsulated in their core, preserving both the nanoparticulate structure and its internal payload. Additionally, the use of Au NPs loaded in PLGA nanoparticles is also an easy and convenient procedure for studying the effectiveness of the payload encapsulation process since it is challenging to know whether each PLGA NP contains rifampicin. In this work, we used the same procedure to load either rifampicin or Au precursors. The role of Au NPs as tracers is also an excellent tool to determine whether the PLGA loading is effective since it is easy to confirm that Au NPs were homogenously distributed inside PLGA NPs produced in this work.

## 5. Conclusions

The developed microfluidic system, formed by coaxial capillaries, allows the controlled production of W/O/W double emulsion droplets that were used as templates of gastro-resistant microparticles based on methacrylic acid-ethyl acrylate copolymers. The stable generation of double emulsion droplets with high monodispersity was successfully achieved by the methodic study of different synthesis variables under flow. As a result, monodisperse spherical microparticles (CV < 5%) loaded with a therapeutic payload (PLGA-RIF NPs) were obtained in a continuous and reproducible fashion. The size of the microparticles can be easily controlled by adjusting a series of parameters that influence the final size of the emulsion droplet, such as the diameter of the capillaries, the viscosity of the two immiscible fluids, the ratio of flow rates between the continuous and middle phases and the surface tension between both. Focusing on the loading function of the Trojan microparticles, using the coaxial microfluidic system, it has been possible to achieve an encapsulation efficiency (EE) close to 100% and a load percentage (DL) of 63%, values much higher than the ones achieved in the discontinuous system, where 49% EE and 7% DL were obtained. Under gastric-simulated conditions (pH = 1.2) we demonstrated that the external Trojan MP coating remained unaltered and that the inner rifampicin-loaded NPs could be rapidly released under intestinal-simulated conditions (pH = 6.8). Consequently, PLGA-RIF would be protected from gastric degradation and potentially reach systemic circulation. We considered the use of PLGA NPs loaded with Au NPs to verify that the Trojan payload is preserved and released depending on the pH. Au-loaded PLGA NPs were used as beacons to demonstrate the successful PLGA NP encapsulation and the release under intestinal-simulated fluid conditions. This work shows great potential concerning loading any type of API in Trojan MPs and would be a reference for future biomedical uses of Trojan MPs.

## Data Availability

Not applicable.

## Supplementary Materials

The following supporting information can be downloaded at: https://www.mdpi.com/article/10.3390/mi13060878/s1, Figure S1: PLGA-RIF calibration; Figure S2: Characterization of the enteric microparticles synthesized in the coaxial microfluidic system with different concentrations of Eudragit.
