# Supplementary material for: Trojan pH-Sensitive Polymer Particles Produced in a Continuous-Flow Capillary Microfluidic Device Using Water-in-Oil-in-Water Double-Emulsion Droplets
Source: Micromachines (Basel). 2022 May 31;13(6):878. doi: 10.3390/mi13060878 (PMC9230220; doi:10.3390/mi13060878)
Supplement: Supplementary file 1 [file micromachines-13-00878-s001.zip › micromachines-1732496-supplementary.pdf]

micromachines

MDPI

Supplementary Materials

Trojan pH-Sensitive Polymer Particles Produced in a Continuous-Flow Capillary Microfluidic Device Using Water-in-Oil-in-Water Double-Emulsion Droplets

Ane Larrea [1], Manuel Arruebo [1,2,3], Christophe A. Serra [4,*] and Victor Sebastián [1,2,3,5,*]

[1] Instituto de Nanociencia y Materiales de Aragón (INMA), CSIC-Universidad de Zaragoza, 50009 Zaragoza, Spain; larrea@unizar.es (A.L.); arruebom@unizar.es (M.A.)
[2] Department of Chemical Engineering, Campus Río Ebro-Edificio I+D, University of Zaragoza, C/Poeta Mariano Esquillor S/N, 50018 Zaragoza, Spain
[3] Networking Research Center on Bioengineering, Biomaterials and Nanomedicine, CIBER-BBN, 28029 Madrid, Spain
[4] Université de Strasbourg, CNRS, ICS UPR 22, F-67000 Strasbourg, France
[5] Laboratorio de Microscopías Avanzadas, Universidad de Zaragoza, 50018 Zaragoza, Spain
* Correspondence: ca.serra@unistra.fr (C.A.S.); victorse@unizar.es (V.S.)

Citation: Larrea, A.; Arruebo, M.; Serra, C.A.; Sebastián, V. Trojan pH-Sensitive Polymer Particles Produced in a Continuous-Flow Capillary Microfluidic Device Using Water-in-Oil-in-Water Double-Emulsion Droplets. *Micromachines* **2022**, *13*, 878. https://doi.org/10.3390/mi13060878

Academic Editor(s): Zebing Mao, Jin Xie and Hong Ding.

Received: date
Accepted: date
Published: 31 May 2022

Publisher's Note: MDPI stays neutral with regard to jurisdictional claims in published maps and institutional affiliations.

Copyright: © 2022 by the authors. Licensee MDPI, Basel, Switzerland. This article is an open access article distributed under the terms and conditions of the Creative Commons Attribution (CC BY) license (https://creativecommons.org/licenses/by/4.0/).

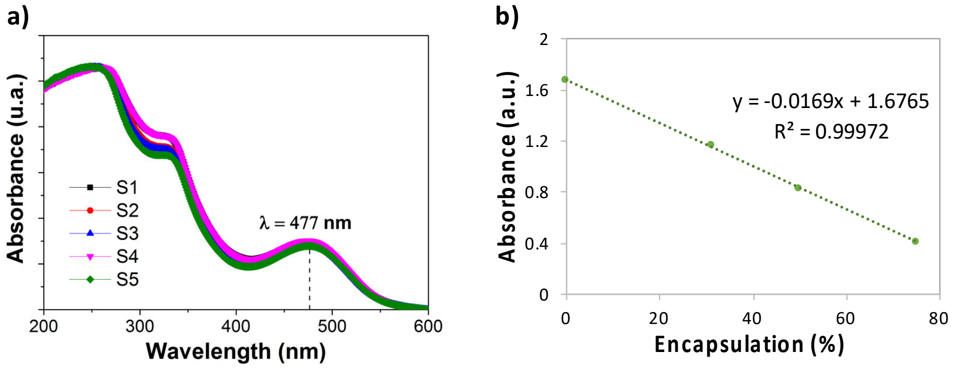

Figure S1. (a) UV-Vis spectra of the NPs released in five different samples of Trojan microparticles. (b) Linear fit obtained from the calibration of PLGA-Rifampicin NPs for determination of %Encapsulation.

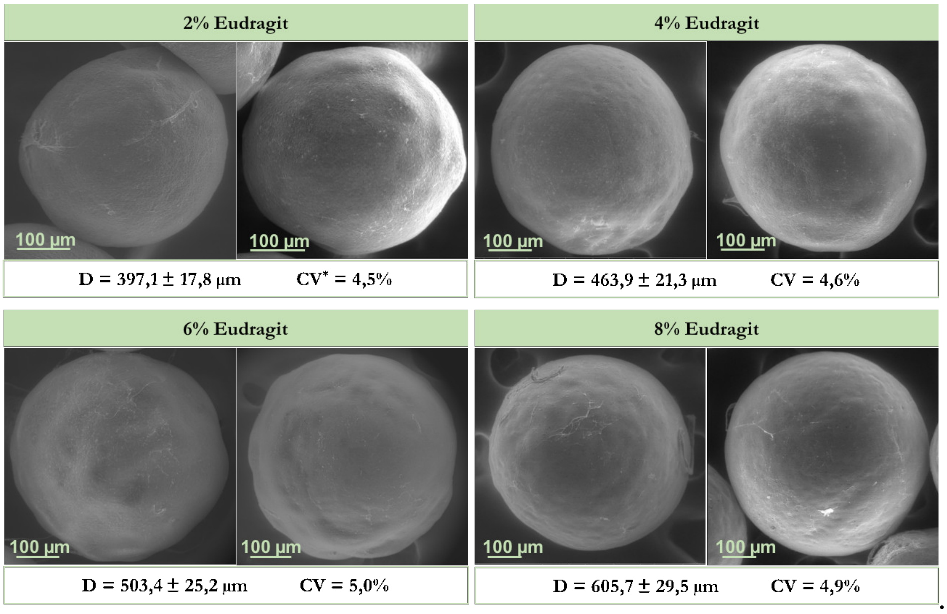

Figure S2. Characterization of the enteric microparticles synthesized in the coaxial microfluidic system with different concentrations of Eudragit. $Q_i/Q_m/Q_o$ = 5/10/400 µl/min. * CV is the ratio between the standard deviation and the average diameter.
